# Prolapsed epiploica of bowel after robotic hysterectomy: A case report

**DOI:** 10.1016/j.amsu.2020.10.055

**Published:** 2020-10-27

**Authors:** Han-Ying Chen, Bor-Ching Sheu, Wen-Chun Chang

**Affiliations:** a Department of Obstetrics and Gynecology, National Taiwan University Hospital and College of Medicine, Taipei, Taiwan

**Keywords:** Robotic assisted hysterectomy, Prolapse, Epiploica, Case report

## Abstract

**Purpose:**

Vaginal cuff dehiscence with evisceration was defined as expulsion of intraperitoneal organs through the separated incision. Prolapsed epiploica of the colon is a rare complication after hysterectomy. The most common organ to prolapsed out from the dehiscence vaginal cuff is terminal ileum. We reported the first known case of prolapsed epiploica of the colon after robotic hysterectomy.

**Case:**

This is a case who had prolapse of a vaginal tumor after sexual intercourse 5 months after robotic hysterectomy. Vaginal tumor resection and primary closure were performed successfully without complications. The final pathology revealed fat prolapse with foreign body reaction and confirmed prolapse of epiploica of the colon. Being aware of the risk factors and patients who are more likely to develop this complication is essential in making the correct diagnosis in time.

**Major conclusion:**

Patients with a higher risk of vaginal cuff dehiscence are advised to avoid sexual activity for a longer period of time. Surgical intervention is the primary treatment. Prolapsed epiploica of the colon should be kept in mind for those who have undergone hysterectomy in order to provide appropriate treatment in time.

## Highlights

• Prolapsed epiploica of the bowel is a rare and potential complication after hysterectomy.
• Higher risk of vaginal cuff dehiscence in early exposure to intercourse.
• Surgical intervention is the primary treatment.
• No definite suggestion on whether vaginal or abdominal approach is superior.

## 1 Introduction

Hysterectomy is one of the most commonly performed gynecological surgery. Vaginal cuff dehiscence and evisceration were first described in 1864 [1]. Both of the events are rare. Vaginal cuff dehiscence with evisceration was defined as expulsion of intraperitoneal organs through the separated incision. The incidence ranged from 0.14 to 4.1% [2,3]. The timing of diagnosis range from as early as three days and as late as 30 years after hysterectomy. The most common organ to prolapsed out from the dehiscence vaginal cuff is terminal ileum, other organs that had been reported including omentum, colon and appendix [2]. The evisceration rarely appears as emergent surgical indication. But emergent surgical intervention is indicated if evisceration appears as bowel incarceration, bowel perforation, and intra-abdominal infection.

The causes of vaginal cuff dehiscence are multifactorial. One of the most common etiology is physical impactions, including sexual intercourse, trans-vaginal probe examination, subsequent vaginal surgery and foreign body insertion. These present mostly in premenopausal women. For menopausal women, the risk factors of vaginal cuff dehiscence include previous vaginal surgery induced scarring tissue, previous radiotherapy, older age, smoker, enterocele repair, and events of sudden increased intra-abdominal pressure. Systemic disease such as poor controlled diabetes mellitus, immune suppressive status or currently receiving chemotherapy treatment may also play roles on poor tissue healing and caused vaginal stump dehiscence. As for the protective factor, obese women (BMI ≥ 30 kg/m2) were 70% less likely than women of normal weight (BMI < 25 kg/m2) to experience vaginal cuff dehiscence after hysterectomy whether it was performed laparoscopically or laparotomy [4].

Due to the limited case amount, there has been no standardized guideline for proper treatment. However, the first-line treatment option that had been carried out in the previous reported cases were primary surgical closure. The approaches may be accessed via transabdominal, transvaginal, laparoscopic or a combination of all above. Most of the cases were treated via vaginal approach. The combine approach via laparoscopic and vaginal route were rare. The first entirely laparoscopic repair of vaginal herniation was reported in 2002 and only accounts for 2% (3 cases) in a large retrospective study that composed of 116 cases [5].

We reported the first known case of prolapsed epiploica of the colon after robotic hysterectomy. This work has been written in line according to the SCARE criteria [6].

## 2 Case report

On May 20, 2019, a 69-year-old woman, gravida 3, para 3, underwent robotic radical cystectomy, total abdominal hysterectomy, bilateral salpingo-oophorectomy and bilateral pelvic lymph nodes dissection for bladder urothelial carcinoma. The vaginal cuff was closed with continuous stitches of absorbable sutures in a two-layer fashion. The postoperative course was uneventful, and the patient was discharged 11 days after surgery. The pelvic MRI on Sep 1, 2019 showed normal wound healing including the vaginal stump (Fig. 1.1 and 1.2).

**Fig. 1:**
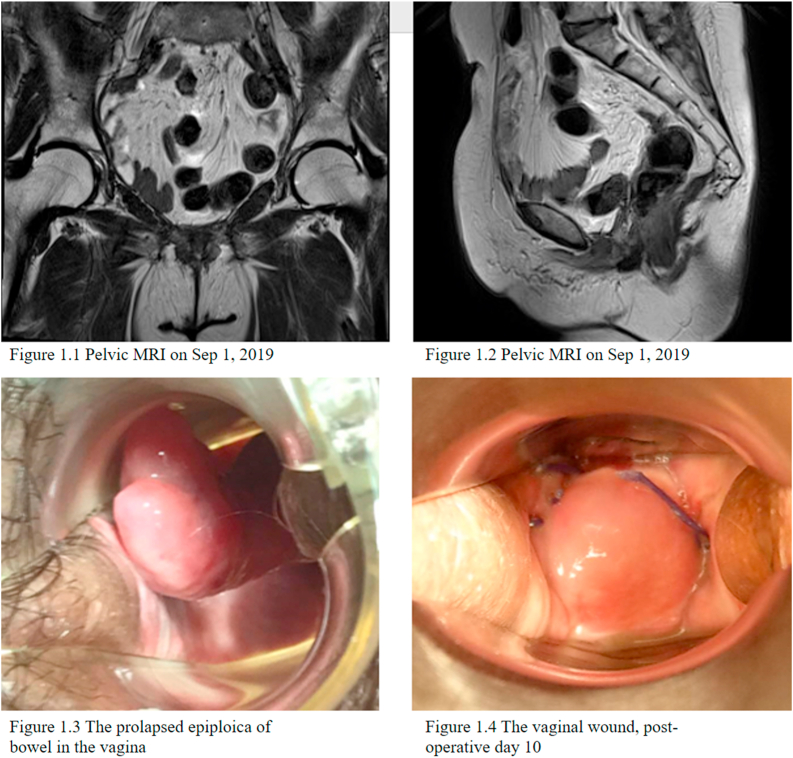
1.1, 1.2: Pre-operative image. 1.3, 1.4: intra-oprative photo.

Five months after the surgery, the patient returned due to bilateral hydronephrosis, suspecting that tumor recurrence was involved. She also complained about a protruding vaginal mass noted after the latest sexual intercourse two weeks ago. The pain gradually increased and the mass was painful upon touching. Speculum examination revealed a 4x3x3cm tubular soft tissue lesion protruding from the anterior aspect of the vaginal apex (Fig 1.3). Considering the patient's history, recurrence of bladder cancer was a possible cause. According to the patient, she had the first postoperative sexual intercourse four months after the surgery without any discomfort. Vaginal tumor excision was arranged 1 week after the patient's return visit, and the vaginal mass shrank to 3 × 2 × 2cm. During the operation, the vaginal tumor was easily removed by a Kelly grasper without the need of cold knife conization or electrocauterization from the vaginal approach. After the soft tissue mass, more likely of prolapsed epiploica (Fig. 1.4), was removed, the vaginal stump ruptured spontaneously. Examination of the vagina showed normal bowel movement and no active bleeding. The dehisced vaginal cuff was closed by interrupted sutures on the first layer and continuous sutures on the second layer with 1-0 Vicryl (polyglactin 910). The total blood loss of the surgery is minimal and no complication is noted. The patient recovered quickly and was able to leave the hospital 24 hours after the operation. The final pathology revealed adipose tissue with inflammatory response which is compatible with the diagnosis of epiploica. On the 30-day post-operative follow up clinic, the vaginal stump is well healed and the patient denied any discomfort in her daily life or during intercourse.

## 3 Discussion

Vaginal cuff dehiscence with evisceration is a potentially devastating complication after hysterectomy due to subsequent bowel ischemia, bowel perforation and peritonitis. Ileum, especially terminal ileum, is the most common site of prolapse [7]. Cases of epiploica with colon and adnexa have been reported [8].

There are several risk factors for vaginal cuff dehiscence, including hysterectomy for malignancy, patients’ health condition such as smoking, malnutrition, and diabetes, and increased abdominal pressure such as constipation, obesity, and chronic coughs [8].

Surgical methods also play an important role in the risk of vaginal cuff dehiscence. A review study included 665 patients who received minimally invasive hysterectomy, and the vaginal cuffs were repaired vaginally, laparoscopically or under robotic assistance [9]. The vaginal cuff dehiscence rate was 0.64% with laparoscopic suturing, 0.18% with vaginal repair and 1.64% with robotic closure. The results are compatible with other previous meta-analyses [3,10]. The reason why robotic closure appears to have higher rates of dehiscence is unclear. Unfamiliar texture feedback with robotic assisted vaginal cuff closure might cause loose sutures, which might be related to the dehiscence rate. However, according to previous studies, the rate declined gradually over the past eight years as robotic surgery became more widely adopted [10]. It is to be noted that vaginal cuff dehiscence frequently occurred after sexual intercourse. In a review [11], the average interval between sexual intercourse and vaginal cuff dehiscence was 7 weeks, which might suggest that a longer period of time is needed before patients can engage in sexual activity. However, there is no study to date that specified the optimal length of pelvic rest. Generally, 6–8 weeks of rest is recommended, and another 4–6 weeks should be advised to those with risk factors or who have already experienced vaginal cuff dehiscence. Initially, patients with vaginal cuff dehiscence would have symptoms such as abdominopelvic pain, vaginal bleeding, mass-like lesion protruding from vagina, increased vaginal discharge or all of the above. Careful physical examination is essential to making the correct diagnosis. Image evaluation with computed tomography is suggested as long as it does not cause a delay in surgical intervention [12]. Surgical intervention is the main treatment, but deciding between transabdominal and vaginal approaches is highly operator-dependent. Laparoscopic repair gives the operator the chance to thoroughly examine the bowel and pelvic condition via a minimally invasive route. Operators with the ability to reduce the bowel transvaginally can choose the transvaginal approach for patients with normal bowel peristalsis and stable vital signs. However, this method is not recommended for patients with peritonitis [8].

## 4 Conclusion

Prolapsed epiploica of the bowel is a rare and potential complication after hysterectomy. Although those with a higher risk of vaginal cuff dehiscence are advised to avoid sexual activity for a longer period of time, there is no randomized control study providing evidence of how long exactly should the pelvic rest last. Surgical intervention is the primary treatment, but there is no definite suggestion on whether vaginal or abdominal approach is superior. Prolapsed epiploica of the colon should be kept in mind for those who have undergone hysterectomy in order to provide appropriate treatment in time.

## Patient consent

The patient has given consent for publication of this case report.

## Provenance and peer review

Not commissioned, externally peer reviewed.

## Author contribution

Han-Ying Chen: Data collection, Data Formal analysis, Data interpretation, Writing the paper. Bor-Ching Sheu: Study concept, Study design, Data collection. Wen-Chun Chang: Study concept and Study design, Writing the paper

## Declaration of competing interest

No potential financial and nonfinancial conflicts is declared.
